# Exploring children’s health literacy in the Netherlands – results in a sample of 8-11 year olds

**DOI:** 10.1093/eurpub/ckac129.320

**Published:** 2022-10-25

**Authors:** J Rademakers, M Hahnraths, T Bollweg, O Okan, M Willeboordse, M Heijmans

**Affiliations:** 1 NIVEL, Utrecht, Netherlands; 2 CAPHRI, Maastricht University, Maastricht, Netherlands; 3 Department of Sport and Health Science, Technical University Munich, Munich, Germany; 4 Mulier Institute, Utrecht, Netherlands

## Abstract

**Background:**

There are few valid, age-appropriate tools to assess children's HL. The German-language European Health Literacy Survey Questionnaire Adapted for Children (HLS-Child-Q15-DE) is a self-report questionnaire adapted from the adult European Health Literacy Survey Questionnaire. In 2021, this instrument was translated and validated in the Netherlands. In this presentation, we will describe the distribution of Health literacy in a sample of Dutch children, and relate their Health literacy level to certain aspects of their health behaviour such as food intake and physical activity.

**Methods:**

The HLS-Child-Q15-DE was translated following WHO guidelines and administered digitally to 209 Dutch schoolchildren (eight-to-eleven-year-olds). Its psychometric properties were assessed and the sample's HL distribution was explored by demographic characteristics. Associations with food intake and physical activity were computed.

**Results:**

Of the sample, 17.2% had a low health literacy score (first quintile), 61.1% medium (second to fourth quintile) and 21.7% high (fifth quintile). Higher HL scores were observed for ten-to-eleven-year-olds (compared with eight-to-nine-year-olds; p = 0.021) and fourth-grade students (compared with third-grade; p = 0.019). A positive association between children's HL and their vegetable consumption and PA behaviour was observed.

**Conclusions:**

Children's health literacy can have an impact on some aspects of their lifestyle. This supports the idea that health literacy evolves throughout life and stresses the importance of both parents and schools in this process.

